# Cartilage from human-induced pluripotent stem cells: comparison with neo-cartilage from chondrocytes and bone marrow mesenchymal stromal cells

**DOI:** 10.1007/s00441-021-03498-5

**Published:** 2021-07-09

**Authors:** Alejandro Rodríguez Ruiz, Amanda Dicks, Margo Tuerlings, Koen Schepers, Melissa van Pel, Rob G. H. H. Nelissen, Christian Freund, Christine L. Mummery, Valeria Orlova, Farshid Guilak, Ingrid Meulenbelt, Yolande F. M. Ramos

**Affiliations:** 1 Dept. of Biomedical Data Sciences, Section Molecular Epidemiology, Leiden University Medical Center, LUMC, Leiden, The Netherlands; 2 Dept. Orthopedic Surgery, Washington University and Shriners Hospitals for Children, St. Louis, USA; 3 Dept. Immunohematology and Blood Transfusion, LUMC, Leiden, The Netherlands; 4 NECSTGEN, Leiden, The Netherlands; 5 Dept. Orthopaedics, LUMC, Leiden, The Netherlands; 6 Dept. Anatomy and Embryology, LUMC, Leiden, The Netherlands; 7 LUMC hiPSC Hotel, Leiden, The Netherlands

**Keywords:** Neo-cartilage, Chondroprogenitor, hiPSCs, Mesenchymal stromal cells, Chondrogenesis, Tissue regeneration

## Abstract

Cartilage has little intrinsic capacity for repair, so transplantation of exogenous cartilage cells is considered a realistic option for cartilage regeneration. We explored whether human-induced pluripotent stem cells (hiPSCs) could represent such unlimited cell sources for neo-cartilage comparable to human primary articular chondrocytes (hPACs) or human bone marrow-derived mesenchymal stromal cells (hBMSCs). For this, chondroprogenitor cells (hiCPCs) and hiPSC-derived mesenchymal stromal cells (hiMSCs) were generated from two independent hiPSC lines and characterized by morphology, flow cytometry, and differentiation potential. Chondrogenesis was compared to hBMSCs and hPACs by histology, immunohistochemistry, and RT-qPCR, while similarities were estimated based on Pearson correlations using a panel of 20 relevant genes. Our data show successful differentiations of hiPSC into hiMSCs and hiCPCs. Characteristic hBMSC markers were shared between hBMSCs and hiMSCs, with the exception of CD146 and CD45. However, neo-cartilage generated from hiMSCs showed low resemblances when compared to hBMSCs (53%) and hPACs (39%) characterized by lower collagen type 2 and higher collagen type 1 expression. Contrarily, hiCPC neo-cartilage generated neo-cartilage more similar to hPACs (65%), with stronger expression of matrix deposition markers. Our study shows that taking a stepwise approach to generate neo-cartilage from hiPSCs via chondroprogenitor cells results in strong similarities to neo-cartilage of hPACs within 3 weeks following chondrogenesis, making them a potential candidate for regenerative therapies. Contrarily, neo-cartilage deposited by hiMSCs seems more prone to hypertrophic characteristics compared to hPACs. We therefore compared chondrocytes derived from hiMSCs and hiCPCs with hPACs and hBMSCs to outline similarities and differences between their neo-cartilage and establish their potential suitability for regenerative medicine and disease modelling.

**Supplementary Information:**

The online version contains supplementary material available at 10.1007/s00441-021-03498-5.

## Introduction

Articular cartilage, the smooth and lubricated tissue lining the end of long bones, plays an important role in mobility by ensuring frictionless articulation while withstanding compressive forces during joint loading. It is composed entirely of chondrocytes, responsible for maintaining tissue homeostasis upon stress, by synthesizing a dense cartilage extracellular matrix (ECM), rich in collagens, proteoglycans, and sulphated glycosaminoglycans (s-GAGs) (Luo et al. 2017; McKee et al. 2019). However, due to a lack of blood supply or lymphatic vessels, cartilage is essentially unable to regenerate, contributing to development of diseases such as osteoarthritis (OA) (Krishnan and Grodzinsky 2018; Patel et al. 2019) and making cartilage regeneration therapies essential to fighting this debilitating condition. Some therapies, based on administering human primary articular chondrocytes (hPACs) and/or mesenchymal stromal cells (MSCs), have been shown to produce stable and healthy neo-cartilage that can be used in implants and for in vitro disease models (de Windt et al. 2017; Ebert et al. 2020; Stenberg et al. 2014).

Previously, we showed the potential of hPAC-derived neo-cartilage for cartilage regeneration based on their 99% similarity of genome-wide methylation to autologous cartilage (Bomer et al. 2016). While autologous neo-cartilage would avoid the immunogenic response that allogenic cells may cause, this technique is relatively invasive for patients since, prior to implantation, a biopsy of the articular cartilage is needed. Alternatively, MSCs can be obtained from several tissues and have the potential to differentiate into relevant cells. Nonetheless, the procedure to obtain them is still invasive, and has a large variability in differentiation efficiency and early senescence in in vitro cultures (Barry 2019; de Windt et al. 2017; Fellows et al. 2016).

Human-induced pluripotent stem cells (hiPSCs) have been proposed to provide an excellent alternative for both cartilage regeneration and disease modeling applications (Liu et al. 2017). Firstly, their production can be scaled, circumventing restrictions in defect size for treatments in the clinic and during disease modeling. Secondly, the use of a cell line circumvents the need for biopsies and thus repeated surgeries on patients. Finally, hiPSCs can be genetically modified to increase chondrogenic potential, introduce patient specific mutations for research purposes, and/or reduce their immunogenicity. Nonetheless, obtaining good quality neo-cartilage from hiPSCs has so far proven challenging.

Issues arise due to the strong variation in differentiation efficiencies between hiPSC lines and clones and a tendency to generate hypertrophic and fibrous matrix (de Windt et al. 2017; Nakayama et al. 2020). Hence, even though several protocols are available, the optimal method for the generation of chondrocytes from hiPSCs remains to be established. Some studies comparing human bone marrow-derived mesenchymal stromal cells (hBMSCs) and hiPSC-derived mesenchymal stromal cells (hiMSCs) suggest major functional and genetic differences, not only between cells but also between neo-cartilage from both cell types (Diederichs and Tuan 2014; Xu et al. 2019). However, in these studies, hiMSCs were generated via the formation of cell aggregates called embryoid bodies (EBs), often variable and with low efficiency (Xu et al. 2019; Diederichs and Tuan 2014), while direct monolayer generation was shown to be more robust (Diederichs et al. 2019).

Alternatively, a stepwise approach could be taken to generate neo-cartilage from hiPSCs via human chondroprogenitor cells (hiCPCs) (Nejadnik et al. 2015; Adkar et al. 2019; Dicks et al. 2020). Notably, differentiation of hiPSCs with this protocol optimizes each developmental step through anterior primitive streak formation and successive emergence of hiCPCs, diminishing variability between independent differentiations. Unfortunately, a major disadvantage of this method is the inefficiency to expand hiCPCs, mainly due to the rapid loss of their chondrogenic potential within a few passages (Adkar et al. 2019).

Here, we aimed to assess upon both approaches towards consistent generation of neo-cartilage from hiPSC with characteristics similar to chondrocytes from hPACS and hBMSCs (the “goldstandard”). We therefore compared chondrocytes derived from hiMSCs and hiCPCs with hPACs and hBMSCs to outline similarities and differences between their neo-cartilage and establish their potential suitability for regenerative medicine and disease modeling.

## Materials and methods

### Sample description and ethics approval

Ethical approval for the RAAK study (Ramos et al. 2014) was obtained from the medical ethics committee of the LUMC (P08.239), and informed consent was obtained from all patients. Approval for the generation of hiPSCs from skin fibroblasts of healthy donors is available under number P13.080.

### Tissue culture and chondrogenesis

#### Cell culture of hiPSCs and primary cells

Two independent control hiPSC lines were used in the current study. Cells were generated from skin fibroblasts of a female: LUMC0030iCTRL12 (030) and a male: LUMC0004iCTRL10 (004) by the LUMC iPSC core facility and registered at the Human pluripotent stem cell registry. Cells were characterized according to pluripotent potential and spontaneous differentiation capacity by the iPSC core facility (Dambrot et al. 2013) and were karyotyped after 15 passages in culture.

hiPSCs were maintained under standard conditions (37 °C, 5% CO_2_) in TeSR-E8 medium (STEMCELL Technologies) on VitronectinXF-coated plates (STEMCELL Technologies). The medium was refreshed daily, and cells were passaged in aggregates using Gentle Cell Dissociation Reagent (STEMCELL Technologies) upon reaching approximately 80% confluency. Human BMSCs and hPACs were collected from OA patients undergoing joint replacement surgery as part of the RAAK study. Collection and expansion of the primary cells has been previously described (Bomer et al. 2016).

#### Differentiation of hiPSC towards hiMSCs and hiCPCs

Human iMSCs were generated using the Stemcell Technologies Mesenchymal Progenitor Kit following the manufacturers’ instructions with small modifications. Following three passages using the recommended Mesencult ACF plus medium, cells were grown in DMEM high glucose (Gibco) supplemented with 10% fetal calf serum (FCS; Biowest), basic FGF (bFGF; 5 ng/ml; Life Technologies), and antibiotics (100 U/ml penicillin, 100 μg/ml streptomycin; Gibco) until elongated and with fibroblast-like morphology. At passage 5, MSC surface markers were analyzed by flow cytometry, and the trilineage potential of the hiMSCs was determined. Generation of hiCPCs was performed as described previously (Adkar et al. 2019). At day 14, analysis for cell surface markers was performed, and hiCPC aggregates were collected for chondrogenesis (Supplementary Fig. S1).

#### Multilineage differentiations

For adipogenesis, 1.5 × 10^4^ cells/cm^2^ were seeded on tissue culture-treated 6-well plates (Cellstar), and differentiation was induced in α-MEM (Gibco) supplemented with 10% FCS, antibiotics, dexamethasone (0.25 μM; Sigma-Aldrich), L-ascorbate-2-phosphate (50 μg/ml; Sigma-Aldrich), insulin (100 μg/ml; Sigma-Aldrich), indomethacin (50 μM; Sigma-Aldrich), and 1-methyl-3-isobutylxantine (0.5 mM; Sigma-Aldrich). Medium was refreshed twice a week for 21 days.

Chondrogenesis was performed in 3D cell pellets following our established protocol (Bomer et al. 2015). In short, cell pellets (hBMSCs, hiMSCs, hPACs) were maintained in DMEM high glucose (Gibco) supplemented with 1% ITS-plus (Corning), dexamethasone (100 nM), L-ascorbate-2-phosphate (50 μg/ml), L-proline (40 μg/ml; Sigma-Aldrich), sodium pyruvate (100 μg/ml; Sigma-Aldrich), TGF-β1 (10 ng/ml; PeproTech), and antibiotics. The medium was refreshed every 3–4 days. Chondrogenesis for hiCPCs was performed basically as described by Dicks et al. (2020): cell aggregates were maintained in DMEM/F-12 (Gibco) supplemented with 1% ITS-plus, 2-Mercaptoethanol (55 μM; Gibco), dexamethasone (100 nM), 1% non-essential amino acids (NEAA; Gibco), L-ascorbate-2-phosphate (50 μg/ml), L-proline (40 μg/ml), TGF-β1 (10 ng/ml), and antibiotics, for 21 days while refreshing medium every 3–4 days. Note that due to their initial stem cell state, hBMSCs and hiMSCs require an extended period for chondrogenesis and deposition of mature cartilage ECM (35 days) as compared to hPACs and hiCPCs (21 days).

Osteogenesis was induced by maintaining day-21 chondrogenic pellets for an additional 14 days with α-MEM supplemented with 10% heat-inactivated FCS, dexamethasone (0.1 μM), L-ascorbate-2-phosphate (50 μg/ml), β-Glycerophosphate (5 mM; Sigma-Aldrich), and antibiotics.

### Flow cytometric analyses

Human BMSCs and hiMSCs were analyzed for the following panel of surface markers: CD31, CD45, CD73, CD90, CD105, CD146, and CD166 (BD Biosciences). hiCPCs were analyzed for CD45, CD90, CD146, and CD166. LIVE/DEAD fixable Aqua Dead Cell stain kit (Thermofisher) was used to define dead cells, and OneComp ebeads (Thermofisher) were used to compensate for the fluorochromes. Data were obtained using the BD LSR-II Flow Cytometer and analyzed with FlowJo 6.0 software.

### RNA isolation and RT-qPCR

Differentiations with hiPSC lines were performed in triplicate. For RNA isolations, two pellets were pooled, and isolation was performed as described previously (Bomer et al. 2015). Total mRNA (150 ng) was processed with a first strand cDNA kit according to the manufacturer’s protocol (Roche Applied Science). cDNA was further diluted five times, and preamplification with TaqMan preamp master mix (Thermo Fisher Scientific Inc.) was performed for a panel of 20 designated genes related to chondrogenesis, hypertrophy, deposition and degradation of cartilage ECM, and neo-cartilage quality (primer sequences in Supplementary Table S1). Gene expression was measured with a Fluidigm Biomark HD machine using a 96.96 IFC chip. Quality control of the data was performed, and non-detected values were imputed according to the minimum detected value. Unsuccessful differentiations, defined by the minimum detected expression of *COL2A1* for hPACs and hBMSCs neo-cartilage, were disregarded.

### Histology and immunohistochemistry

Tissues (neo-cartilage and neo-bone) were fixed in 4% formaldehyde and embedded in paraffin. After sectioning, slides were deparaffinized and rehydrated prior to histology or immunohistochemistry.

Overall cellular and tissue structure was visualized with hematoxylin–eosin (HE) staining. Glycosaminoglycans were visualized by staining with 1% Alcian Blue 8-GX (Sigma-Aldrich) and Nuclear Fast red staining (Sigma-Aldrich). Calcium deposits were stained with 2% Alizarin Red S (Sigma-Aldrich).

To detect COL2 (MAB1330; Millipore; 1:100 in TBST/10% normal goat serum, overnight at 4 °C), COL1 (ab34710; Abcam; 1:1000 in TBST/10% normal goat serum, overnight at 4 °C), and COL10 (× 53/2031501005; Quartett; 1:100 in TBST/10% normal goat serum, overnight at 4 °C), immunohistochemistry was performed with 3-diaminobenzidine (DAB) solution (Sigma-Aldrich) and hematoxylin (Klinipath) as described before (Bomer et al. 2015).

Lipid droplets were stained for 10 min with Oil-Red-O solution (Sigma-Aldrich) after fixation of the cells in 4% formaldehyde. To reduce the background, the following staining cells were gently washed with 60% isopropanol and distilled water.

### Statistics and similarities

Relative gene expression (−ΔCt values) was calculated using levels of glyceraldehyde 3-phosphate dehydrogenase (*GAPDH*) and acidic ribosomal phosphoprotein P0 (*ARP*) as housekeeping genes. Betas, standard errors (SE), and *P*-values for gene expression differences across cell types were determined by applying generalized estimation equations (GEE; IBM SPSS software). *P* < 0.05 were considered statistically significant.

Similarities between the different cell types and differentiations were calculated based on Pearson correlations using a panel of 20 relevant genes.

## Results

### Generation and characterization of hiMSCs

**Fig. 1:**
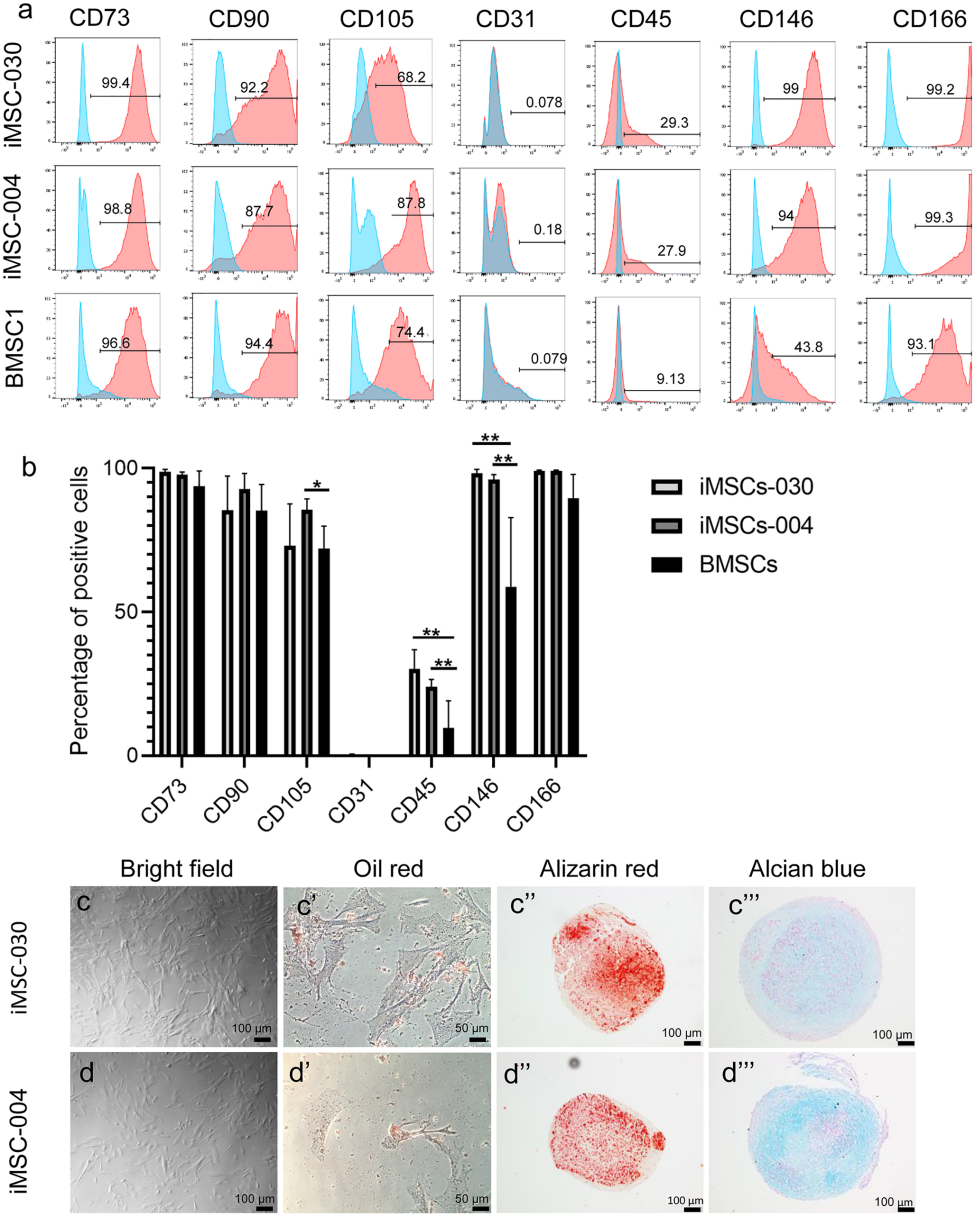
Characterization of hiMSCs*.* (**a**–**b**) Flow cytometry analysis of MSC characteristic markers. The blue histogram shows unstained cells, while the red histogram shows specific marker cell staining. Results shown are the average of three independent differentiations with their standard deviation for each hiPSC line and for three hBMSC lines (hiMSC-030: CD146: **P = 3.1 × 10^−7^ and CD45, **P = 5.9 × 10^−10^; hiMSC-004: CD146, **P = 1.4 × 10^−6^; CD45, **P = 1.0 × 10^−30^ and CD105, *P = 4.2 × 10^−4^). (**c**–**d**) Bright field microscopy image of hiMSCs and representative images for trilineage differentiation. Human iMSCs show a fibroblastic and spindle-shaped morphology; adipocytes were stained by Oil red (**c**′–**d**′), osteocytes by Alizarin red (**c**″–**d**″), and chondrocytes by Alcian blue (**c**‴–**d**‴)

Two independent control hiPSC lines, well-characterized by morphology, pluripotent status, spontaneous differentiation capacity, and by karyotyping, were used for this study (Supplementary Fig. S2; Dambrot et al. 2013). Cells were differentiated towards hiMSCs and compared to hBMSCs after five passages. Expression of typical MSC surface markers as defined by the International Society of Cellular Therapy (ISCT: presence of CD73, CD90, CD105; absence of CD31, CD45 (Dominici et al. 2006)) and expression of CD146 and CD166 (expressed in chondroprogenitor cells (Dicks et al. 2020)) were assessed by flow cytometry (Fig. 1(a–b)). Highly comparable expression was observed for CD73, CD90, CD105, and CD166 between hiMSCs and hBMSCs, while cells were negative for CD31. Significant differences, however, were found for CD146 and CD45. Both markers were expressed in a larger percentage of the hiMSC population compared to 44% and 9% in hBMSCs, respectively (CD146: for hiMSC-030 and hiMSC-004 resp. 98% and 96%, *P* = 3.1 × 10^−7^ and 1.4 × 10^−6^; CD45: for hiMSC-030 and hiMSC-004 resp. 29% and 28% with *P* = 5.9 × 10^−10^ and 1.0 × 10^−30^). Figure 1(c–d) shows morphology of hiMSCs, with majority of the cells being spindle-shaped, elongated, and fibroblast-like. Importantly, hiMSCs showed tri-lineage differentiation into fat (Oil red; Fig. 1(c′–d′)), bone (Alizarin red; Fig. 1(c″–d″)), and cartilage (Alcian blue; Fig. 1(c‴–d‴)), as confirmed by histology. Altogether, our analyses confirmed successful differentiation of hiPSCs into a mesenchymal stromal cell type.

### Generation and characterization of hiCPCs

**Fig. 2:**
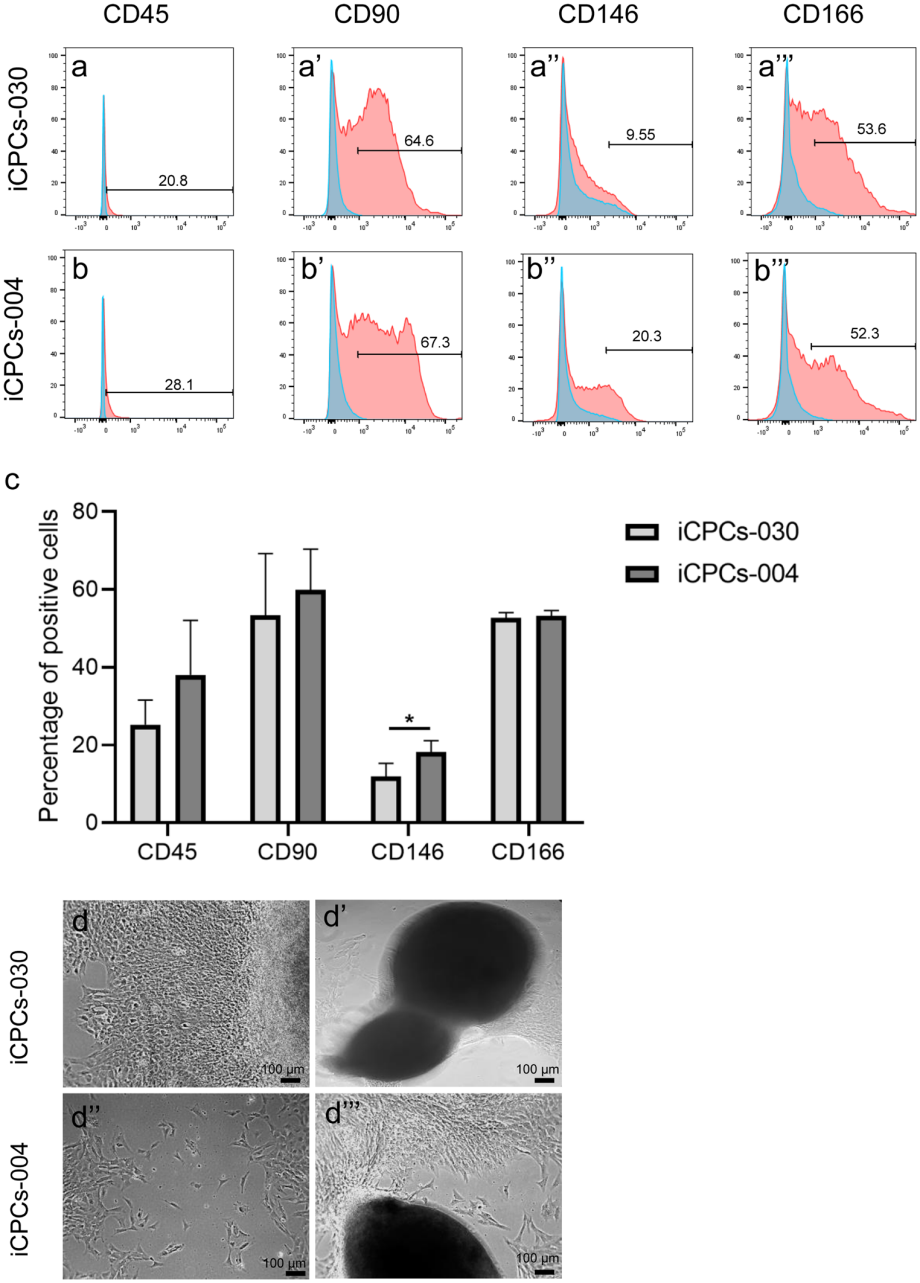
Characterization of hiCPCs. (**a**, **b**, **c**) Flow cytometry analysis of CD45, CD90, CD146, and CD166 for hiCPCs. Results shown are the average of independent differentiations for each hiPSC line (*n* = 2, *P = 5.1 × 10^−3^). (**d**) Bright field microscopy image of hiCPCs showing cells growing in monolayer and cell aggregates following 14 days of differentiation

Control hiPSCs were differentiated towards hiCPCs. After 14 days, analysis of cell surface markers showed similar expression of CD45, CD90, and CD166 across both hiPSC lines (Fig. 2(a, b, c)). However, CD146 was expressed within a lower percentage of hiCPC-030 as compared to hiCPC-004 (10% versus 20%, *P* = 5.1 × 10^−3^). Notably, overall percentages of CD90, CD146, and CD166 positive cells appeared smaller than compared to the hiMSCs, while the percentage of CD45-positive hiCPCs was relatively large (38% and 25% among hiCPCs-004 and hiCPCs-030, respectively). Figure 2(d) shows cell morphology, indicating population heterogeneity and spontaneous cell aggregation during the hiCPC-generating process.

### Histochemistry analysis of neo-cartilage

**Fig. 3:**
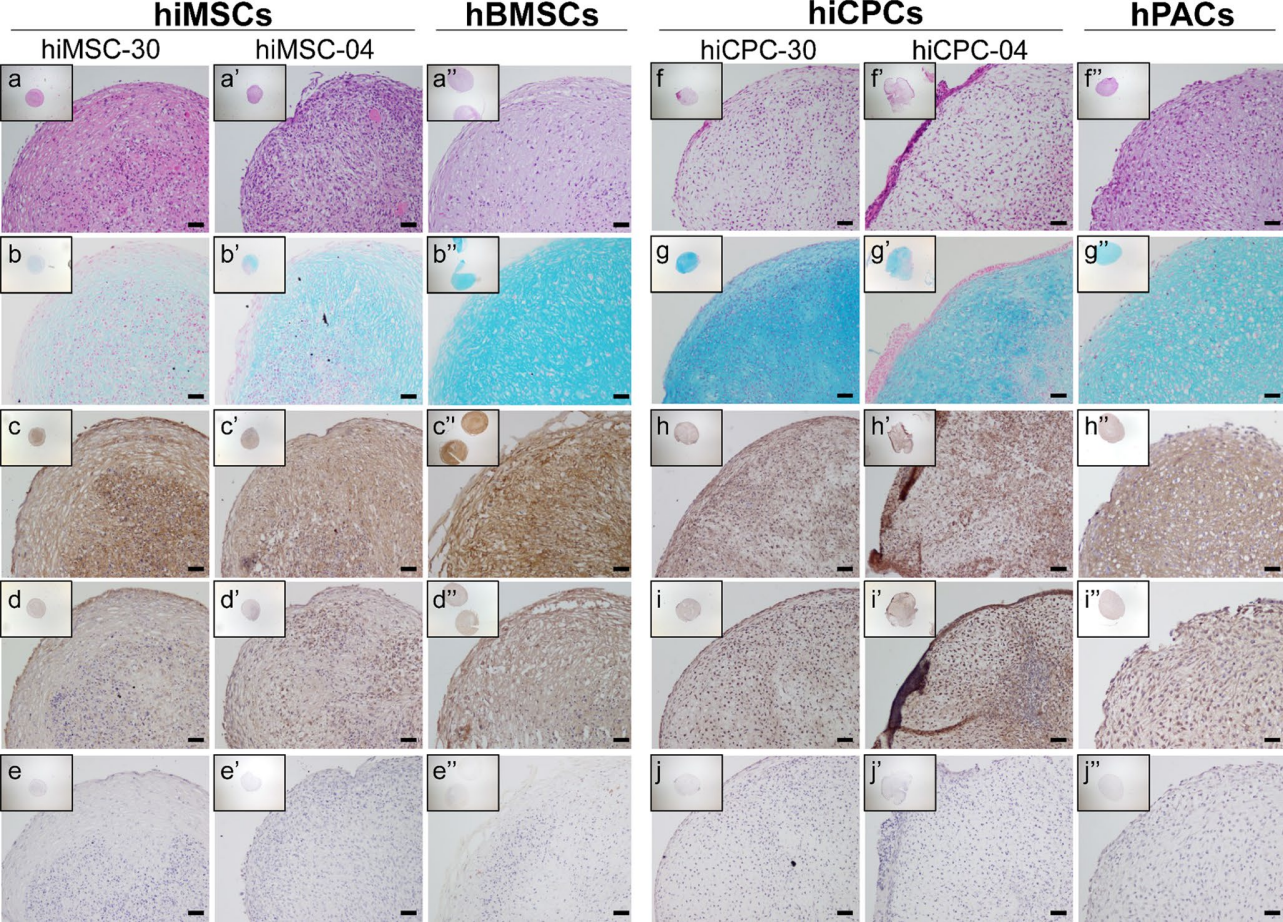
Histology and immunohistochemistry of neo-cartilage. Representative images of neo-cartilage generated by hiMSCs and hBMSCs after 35 days of chondrogenesis (**a**–**e**), or by hiCPCs and hPACs following 21 days of chondrogenesis (**f**–**j**), stained with HE (**a** and **f**), Alcian Blue (**b** and **g**), COL1 (**c** and **h**), COL2 (**d** and **i**), and COL10 (**e** and **j**). Scale bars: 50 μm

Prior to quantitative gene expression analyses, general neo-cartilage pellet formation and cellular structures of hiMSCs and hiCPCs were compared to that of hBMSCs and hPACs by HE and Alcian Blue staining. Following 35 days of chondrogenesis, HE staining of hiMSC neo-cartilage showed the presence of a core with higher number of cells, concurrent with less matrix as compared to hBMSC-derived neo-cartilage (Fig. 3(a–f)). Yet, the presence of lacunae can be observed in the hiMSC neo-cartilage, indicating successful generation of cartilage ECM as also confirmed by the Alcian Blue staining (Fig. 3(b–g)). To reduce heterogeneity of hiCPC population, 3D pellets were generated starting from cell aggregates (such as indicated in Fig. 2(d–d′)). HE staining showed relatively homogeneous ECM deposition, lacunae formation, but also off-target cells on the outer surface of some hiCPC pellets (Fig. 3(f′); hiCPC-004). When comparing hiCPC- and hPAC-derived neo-cartilage, Alcian Blue staining seemed more intense and homogenous as compared to that of hiMSCs and hBMSCs (compare Fig. 3(b–b″ and g–g″)).

### Gene expression profiles and immunohistochemistry of hiMSC-, hBMSC-, and hPAC-derived neo-cartilage

**Fig. 4:**
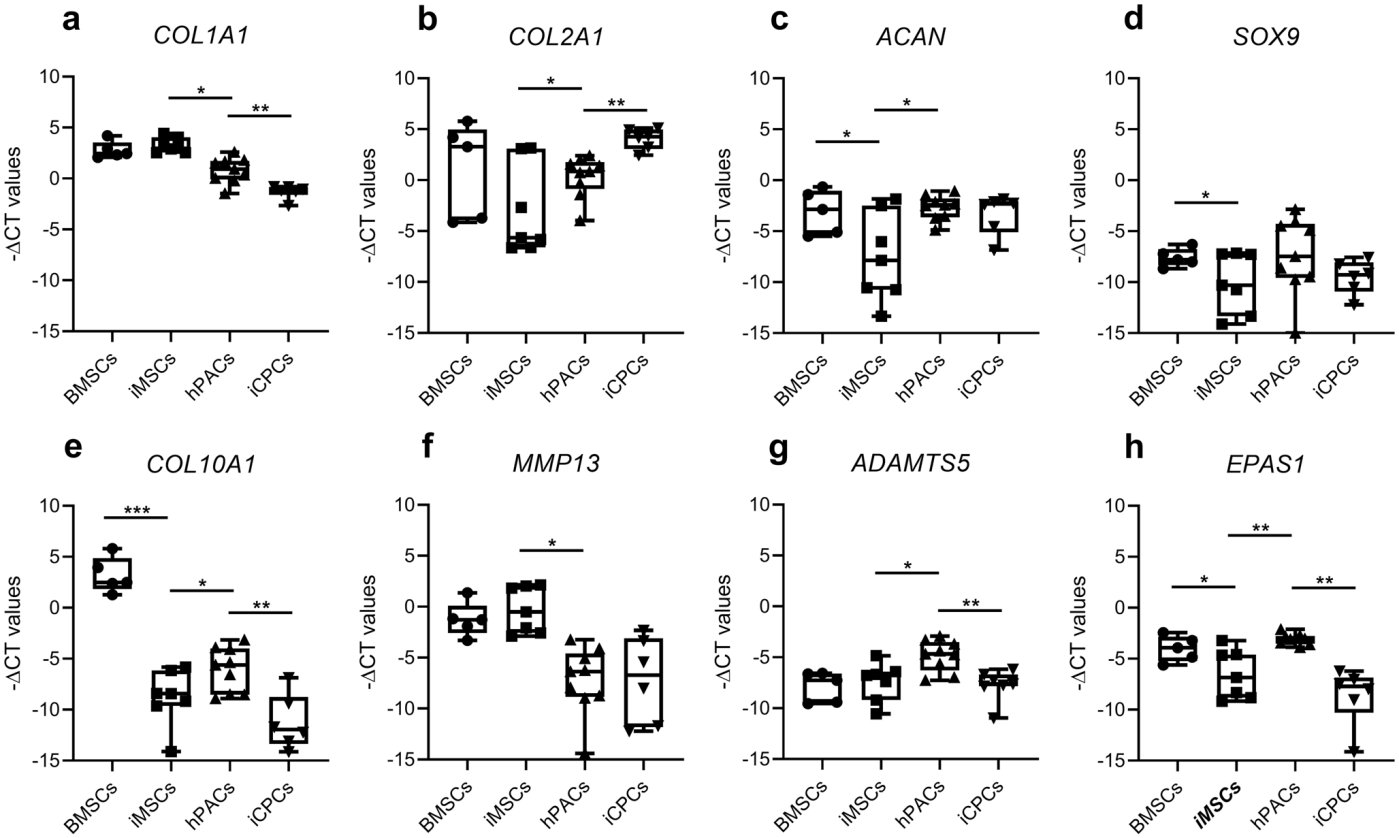
Boxplots for −ΔCt values of matrix, hypertrophy, and chondrogenic genes (**a**–**h**) as indicated for hiMSCs and hBMSCs, and hiCPCs and hPACs, following 35 days (hBMSCs, hiMSCs) and 21 days (hPACs, hiCPCs) of chondrogenesis (*n* = 5–7; *P < 0.05; **P < 10^−4^; ***P < 10^−6^)

To characterize chondrogenesis efficiency, RT-qPCR was performed of hiMSC- and hBMSC-derived neo-cartilage (day 35) and hPAC-derived neo-cartilage (day 21). Fold differences were calculated for chondrocyte-specific genes relative to hBMSC-derived neo-cartilage (Table 1; Fig. 4). While the expression of *COL2A1* only showed a trend towards lower expression (FD =  −17.2, *P* = 9.0 × 10^−2^), significantly lower levels of matrix gene *ACAN* (FD =  −21.8, *P* = 1.1 × 10^−2^) and chondrogenic transcription factor *SOX9* (FD =  −3.9, *P* = 2.6 × 10^−2^) were expressed in hiMSC-derived neo-cartilage compared to that from hBMSCs. Additionally, in hiMSC-derived neo-cartilage, *EPAS1* was significantly lower (FD =  −5.7, *P* = 9.8 × 10^−3^), and hypertrophic cartilage marker *COL10A1* was very lowly expressed (FD =  −4092.3, *P* = 1.0 × 10^−30^).

**Table 1:** Differences in gene expression between hiMSC-and hBMSC-derived neo-cartilage at week 5 of chondrogenesis

hiMSCs versus hBMSC neo-cartilage				
	Fold difference	Beta	SE	P value
Matrix genes				
*ACAN*	**−21.8**	**−4.4**	**1.7**	**1.1E − 02**
*COL2A1*	−17.2	−4.1	2.4	9.0E − 02
*COL1A1*	1.4	0.5	0.4	2.7E − 01
*COL10A1*	**−4092.3**	**−12.0**	**1.2**	**1.0E − 30**
Hypertrophy genes				
*ADAMTS5*	1.4	0.5	0.9	5.9E − 01
*MMP13*	2.0	1.0	1.0	3.5E − 01
*EPAS1*	**−5.7**	**−2.5**	**1.0**	**9.8E − 03**
*WWP2*	−1.2	−0.3	0.3	2.8E − 01
*ALPL*	−3.1	−1.6	1.3	2.1E − 01
Chondrogenesis genes				
*SOX5*	−3.9	−2.0	1.3	1.3E − 01
*SOX6*	−2.2	−1.1	0.9	2.2E − 01
*SOX9*	**−3.9**	**−2.4**	**1.1**	**2.6E − 02**
*FGFR2*	**−22.0**	**−4.5**	**1.6**	**5.9E − 03**
*NOTCH1*	1.5	0.6	0.8	5.0E − 01
*NOTCH3*	−2.9	−1.5	0.9	6.9E − 02
*SMAD3*	1.4	0.5	0.5	3.3E − 01
*SMAD7*	1.0	0.0	0.5	9.7E − 01
*GDF5*	1.6	0.2	0.5	6.4E − 01
*PRG4*	−5.0	−0.4	0.7	6.0E − 01
*NFAT5*	**−1.5**	**−0.6**	**0.3**	**1.9E − 02**

Significant differential expression depicted in bold

Based on the gene expression profiles, we determined that following 35 days of chondrogenesis, neo-cartilage pellets derived from hiMSCs and hBMSCs were 53% similar (SD = 16; see Supplementary Table S2a for complete overview of hiMSC-hBMSC similarities). Since the similarity was not very strong, we questioned whether differentiated hiMSCs were more comparable to hPACs. However, based on the expression profile of our gene panel, we found only 39% similarity (SD = 20; see Supplementary Table S2c for a complete overview of hiMSC-hPAC similarities). In fact, the majority of the genes here assessed (14 out of 20; Table 2) were significantly different expressed between hiMSC- and hPAC-derived neo-cartilage. Specifically, expression of matrix genes such as *COL2A1* (FD =  −10.5, *P* = 4.2 × 10^−2^) and *ACAN* (FD =  − 29.5, *P* = 7.6 × 10^−3^) were lower, while catabolic and mineralization genes such as *MMP13* (FD = 123.2, *P* = 1.4 × 10^−3^), *COL1A1* (FD = 5.5, *P* = 1.7 × 10^−3^), and *ALPL* (FD = 51.7, *P* = 1.4 × 10^−3^) were higher expressed. Altogether, this suggests that during chondrogenesis, hiMSCs deposit neo-cartilage of inferior quality as compared to that of hPACs.

**Table 2:** Differences in gene expression between hiMSC- and hPAC-derived neo-cartilage at respectively weeks 5 and 3 of chondrogenesis

hiMSCs versus hPAC neo-cartilage				
	Fold difference	Beta	SE	P value
Matrix genes				
*ACAN*	**−29.5**	**−4.9**	**1.6**	**7.6E − 03**
*COL2A1*	**−10.5**	**−3.4**	**1.7**	**4.2E − 02**
*COL1A1*	**5.5**	**2.5**	**0.5**	**1.7E − 03**
*COL10A1*	**−6.7**	**−2.8**	**1.2**	**2.0E − 02**
Hypertrophy genes				
*ADAMTS5*	**−5.9**	**−2.6**	**0.8**	**1.8E − 03**
*MMP13*	**123.2**	**6.9**	**1.3**	**1.4E − 03**
*EPAS1*	**−10.9**	**−3.4**	**0.8**	**4.8E − 05**
*WWP2*	**−2.3**	**−1.2**	**0.4**	**2.8E − 03**
*ALPL*	**51.7**	**5.7**	**1.8**	**1.4E − 03**
Chondrogenesis genes				
*SOX5*	**−8.2**	**−3.0**	**1.3**	**2.3E − 02**
*SOX6*	−2.6	−1.4	0.9	1.5E − 01
*SOX9*	−5.4	−2.4	1.7	1.4E − 01
*FGFR2*	**−89.6**	**−6.5**	**1.5**	**2.1E − 05**
*NOTCH1*	−1.1	−0.1	0.7	8.6E − 01
*NOTCH3*	2.0	1.0	0.7	1.7E − 01
*SMAD3*	**−2.4**	**−1.3**	**0.6**	**2.5E − 02**
*SMAD7*	1.6	0.7	0.6	2.1E − 01
*GDF5*	**−22.0**	**−0.7**	**0.2**	**1.8E − 04**
*PRG4*	**−77.7**	**−1.1**	**0.1**	**3.5E − 09**
*NFAT5*	−1.2	−0.2	0.3	4.3E − 01

Significant differential expression depicted in bold

Although inherently less sensitive to gene expression levels, hence less suitable for quantitative analyses, immunohistochemistry of COL1, COL2, and COL10 was performed to allow visualization of protein localization for hBMSC- and hiMSC-derived neo-cartilage. As it can be seen in Fig. 3(c–c″), COL1 in hiMSC-derived neo-cartilage seemed to be particularly localized in the surrounding of cells and at the core of the neo-cartilage pellet, while BMSC-derived neo-cartilage showed a homogeneous staining across the matrix. COL2 staining of hiMSC-derived neo-cartilage as compared to BMSC-derived neo-cartilage showed more variability, while being particularly localized, across all the different cell lines, in the cytoplasm and not in the ECM (Fig. 3(d–d″)). With respect to COL10A1 protein expression, staining intensity was generally low similar to the *COL10A1* gene expression (Fig. 3(e–e″)).

### Characterization of differences between hiCPC- and hPAC-derived neo-cartilage

Subsequently, hiCPC chondrogenesis was characterized. In contrast to hBMSCs, hiCPCs already showed a strong deposition of cartilage ECM at day 21 as determined by Alcian Blue and COL2 staining (Fig. 3(g–g″ and i–i″)). Furthermore, we noticed that based on expression levels of *COL2A1*, 79% of all hiCPC-derived pellets passed our criterium for deposition of neo-cartilage. Among hiMSC differentiations, however, more variation was observed and fewer pellets (54%) passed the pre-set threshold for expression levels of *COL2A1*.

Gene expression analyses of hiCPC-derived neo-cartilage compared to that of hPACs (Table 3; Fig. 4) demonstrated significantly higher levels of *COL2A1* (FD = 13.0, *P* = 5.7 × 10^−7^) and lower expression of genes associated with cartilage hypertrophy, such as *COL10A1* (FD =  −35.9, *P* = 5.7 × 10^−7^) and *COL1A1* (FD =  −4.3, *P* = 7.7 × 10^−6^). In addition, levels of the catabolic gene *ADAMTS5* were significantly lower (FD =  −5.2, *P* = 1.0 × 10^−5^). Together, this indicates enhanced quality of matrix deposited by hiCPCs during chondrogenesis. Comparison of the chondrocyte-specific gene panel showed 65% similarity (SD = 12.5) between hiCPC- and hPAC-derived neo-cartilage (see Supplementary Table S2b for complete overview of hiCPC-hPAC similarities). Prolonged chondrogenesis of hiCPCs until day 35 did not further improve similarity with hPACs, while expression levels of hypertrophic and mineralization gene *ALPL* significantly increased (FD = 4.0, *P* = 1.8 × 10^−2^; Supplementary Table S3).

**Table 3:** Differences in gene expression levels between hiCPC- and hPAC-derived neo-cartilage at week 3 of chondrogenesis

hiCPCs versus hPAC neo-cartilage				
	Fold difference	Beta	SE	P value
Matrix genes				
*ACAN*	−1.6	−0.7	0.8	4.2E − 01
*COL2A1*	**13**	**3.7**	**0.7**	**5.7E − 07**
*COL1A1*	**−4.3**	**−2.1**	**0.5**	**7.7E − 06**
*COL10A1*	**−36**	**−5.2**	**1.2**	**1.9E − 05**
Hypertrophy genes				
*ADAMTS5*	**−5.2**	**−2.4**	**0.5**	**1.0E − 05**
*MMP13*	1.0	0.1	1.9	9.7E − 01
*EPAS1*	**−48**	**−5.6**	**1.1**	**2.1E − 07**
*WWP2*	1.0	0.0	0.5	9.6E − 01
*ALPL*	1.8	0.8	1.8	6.4E − 01
Chondrogenesis genes				
*SOX5*	1.4	0.5	0.4	2.4E − 01
*SOX6*	−2.3	−1.2	1.4	3.9E − 01
*SOX9*	−3.8	−1.9	1.5	1.9E − 01
*FGFR2*	1.5	0.6	0.5	2.8E − 01
*NOTCH1*	3.1	1.6	0.9	5.7E − 02
*NOTCH3*	1.7	0.8	0.6	2.1E − 01
*SMAD3*	**−8.7**	**− 3.1**	**1.0**	**1.2E − 03**
*SMAD7*	−1.9	−0.9	1.4	5.0E − 01
*GDF5*	**−15.7**	**−1.3**	**0.3**	**5.0E − 06**
*PRG4*	**−18.3**	**−0.8**	**0.2**	**1.0E − 06**
*NFAT5*	−1.2	−0.3	0.3	3.7E − 01

Significant differential expression depicted in bold

To explore protein localization and matrix structure, COL1, COL2, and COL10 staining was performed for hiCPC- and hPAC-derived neo-cartilage pellets. As can be observed in Fig. 3(h″), COL1 staining was consistently expressed throughout the ECM of the hPAC-derived neo-cartilage, while hiCPC-derived pellets (Fig. 3(h)) showed a less uniform staining. Expression of COL2 was well-detectable in the hiCPC neo-cartilage throughout the pellets and comparable to hPAC-derived neo-cartilage (Fig. 3(i–i″)). Comparable to hBMSC- and hiMSC-derived neo-cartilage, only faint COL10 expression in the ECM was observed (Fig. 3(j and j″)).

## Discussion

To get more insight into the consistency of frequently used neo-cartilage differentiation protocols for hiPSCs, as well as the resulting neo-cartilage quality, we here compared a stepwise protocol to generate human chondroprogenitor cells (hiCPCs) and hiPSC-derived mesenchymal stromal cells (hiMSCs), then allowed them to undergo chondrogenesis in parallel with human primary chondrocytes (hPACs) and bone marrow mesenchymal stromal cell (hBMSCs) equivalents. The results obtained with our 20-gene chondrocyte-specific gene panel showed almost 70% similarity of hiCPC neo-cartilage when compared with human primary chondrocytes. This stepwise protocol circumvented the need for intermediate cells (hiMSCs), for which we found only 39% similarity to hPACs. In addition to the relatively high similarity, the advantages of the stepwise approach include the shorter time frame and high efficiency of chondrogenesis.

Based on a pre-set threshold for expression levels of *COL2A1*, 79% of the hiCPC pellets deposited good neo-cartilage, while, in line with previous studies (Diederichs et al. 2019; Diederichs and Tuan 2014), chondrogenesis with the hiMSCs was successful in 54% of the pellets. Among others, hiCPC-derived neo-cartilage showed significantly (13-fold) higher expression of *COL2A1* compared to that from hPACs, which was in accordance with the COL2 protein expression as detected with immunohistochemistry. *COL1A1* and *COL10A1* expression were 4.3-fold and 36-fold lower, respectively, than their levels in hPACs. Results of COL1 immunohistochemistry were in line with this; however, for COL10 expression, we did not observe pronounced differences across the different cell sources. Furthermore, the expression level of *ADAMTS5* in hiCPC-derived neo-cartilage was found to be 5.2-fold lower than that in hPACs, which may explain the visibly higher Alcian blue intensity, indicative of s-GAG levels in the hiCPC-derived neo-cartilage. Together, our data denote that generation of hiCPC-derived neo-cartilage offers promising prospects for skeletal regenerative therapies with less hypertrophic neo-cartilage, although further improvement in differentiation efficiency and quality may still be possible and further confirmation of applicability by in vivo experiments will be required.

Unfortunately, a major disadvantage of hiCPCs is the reduction of their chondrogenic potential following expansion in vitro (Adkar et al. 2019; Dicks et al. 2020), requiring repeated chondrogenic differentiations to ensure deposition of high quality neo-cartilage. A possible culprit of this is the generation of a diverse heterogenous hiCPC population, where neurogenic and mesenchymal lineage cells are involved (Dicks et al. 2020; Wu et al. 2021). A chondrogenic selection of this population and further optimization of differentiation factors may improve chondrogenic potential and diminish expansion problems while increasing cartilage quality. Such increase in differentiation potential has been demonstrated by Dicks et al. when sorting for CD146, CD166, and PDGFR $\beta$ surface marker expression or by using a GFP-COL2A1 reporter hiPSC line. This COL2A1 marker, however, is known to be expressed in a wide variety of tissues (Seufert et al. 1994). Therefore, another option would be to use a reporter line with an earlier chondrogenic marker, such as *SOX9*, to further enhance the efficiency of the differentiation. This was recently performed for immortalized adipose-derived stem cells with stable *SOX9* overexpression, which showed enhanced chondrogenic potential (Katz et al. 2020).

Of note was the expression of CD45 in both hiCPC lines (38% of hiCPC-004 with SD = 14 and 25% of the hiCPC-030 with SD = 6.3) since CD45 is a transmembrane protein tyrosine phosphatase and a known characteristic of hematopoietic cells (Fellows et al. 2017). It has been found that chondrogenesis in the presence of CD45-positive cells of hematopoietic origin enhanced the expression of chondrogenic genes such as *COL2A1* and *SOX9* (Kuznetsov et al. 2001). Therefore, the CD45-expressing cells within the mixed population of cells from different lineages that are generated with the stepwise protocol may contribute to enhancing the chondrogenic potential of the cells. This was, however, not observed for the hiMSCs.

Characterization of the hiMSCs showed that the well-known hBMSC surface markers (i.e., CD90, CD105, CD73, CD31, CD166) were similarly expressed across the various differentiations, with exception of CD45 (27% of hiMSCs with SD = 6 as compared to 10% of hBMSCs with SD = 9) and CD146 (97% of hiMSCs with SD = 2 as compared to 59% of hBMSCs with SD = 24). CD146 is a transmembrane glycoprotein that belongs to the immunoglobulin superfamily of cell adhesion molecules (CAMs), and is involved in cell adhesion and proliferation (Buchert et al. 2019). Furthermore, it has been described as an excellent multipotency marker for MSCs, as compared to specialized cells (Espagnolle et al. 2014; Harkness et al. 2016; Matta et al. 2019), while showing a direct correlation to chondrogenic potential (Su et al. 2015).

Comparison of hiMSC- and BMSC-derived neo-cartilage showed a 53% similarity. Although this is considerable, it should be noted that the hiMSCs from both hiPSC lines and across all differentiations performed do display high levels of heterogeneity, as shown in Fig. 3. To compensate for this, Diederichs et al. suggested pre-selecting cells with high expression levels of *SOX9* after a week in culture (Diederichs et al. 2019). In their study, this approach increased the success rate and reduced variation. On the other hand, as also observed before (Diederichs et al. 2019), *COL10A1* was very lowly expressed at gene expression and protein level, which is characteristic of poor neo-cartilage ECM. Improvement may be established by modifications of the chondrogenic medium, such as by adding BMP2 or BMP4 (Xu et al. 2019). Finally, when comparing hiMSC- and hPAC-derived neo-cartilage, we can strongly conclude that matrix generated by hiMSC has a hypertrophic phenotype with a 39% similarity to neo-cartilage from primary chondrocytes. This is defined by the lower expression of *COL2A1*(−10.5 fold lower), while *COL1A1*, *ALPL*, and *MMP13* were highly upregulated (5.5, 51.7, and 123.2-fold, respectively). The expression of *MMP13* and *ALPL* would suggest a higher collagen degradation with a subsequent calcification, characteristic of terminal chondrogenic differentiation, endochondral ossification, and OA initiation (Chen et al. 2015; Li et al. 2017). Quantification of MMP13 enzymatic activity could help to determine whether the gene expression upregulation also results in an increase of the activate protein (Li et al. 2017). The observed differences in neo-cartilage were expected since neo-cartilage from BMSCs and hPAC have a low similarity, and it could be advocated that hiMSCs are an ideal candidate for studying skeletal diseases in which endochondral bone formation and hypertrophy are a driving mechanism (Dreier 2010; Kerkhofs et al. 2016).

Although hPACs were collected from macroscopically unaffected regions of the articular cartilage, a potential drawback of our study is that they were collected from patients undergoing joint replacement surgery due to end stage OA. Hence, it could be speculated that given the higher *COL2A1* and concurrent lower *COL1A1* and *ADAMTS5* levels in hiCPC-derived neo-cartilage, hiCPCs deposit neo-cartilage that is more comparable to healthy cartilage. However, the acquisition of healthy tissue is a challenge in the field, and potential differences between hPACs from preserved and healthy cartilage remain to be determined. Additionally, the emphasis of our manuscript is on the sensitive signaling processes occurring during chondrogenesis. Consequently, further analysis of other significantly different genes and other intrinsic chondrogenic mechanisms would still need to be confirmed by protein expression and ultimately tested in an in vivo model.

## Conclusion

When taking a stepwise approach for chondrogenesis from hiPSCs via chondroprogenitor cells, similarities of almost 70% to primary chondrocytes can be accomplished within 21 days of chondrogenesis. For application of regenerative therapies, this may well be very promising. On the other hand, chondrogenesis methods via hiMSCs result in lower similarity to hPACs, while levels of hypertrophic markers are higher. As such, hiMSCs may be more suitable for in vitro models of skeletal diseases.

## Supplementary Information

Below is the link to the electronic supplementary material.

Supplementary file1 (DOCX 2286 KB)

## Data Availability

The data that support the funding of this study are available from the corresponding author upon request.
