# Supplementary material for: Cartilage from human-induced pluripotent stem cells: comparison with neo-cartilage from chondrocytes and bone marrow mesenchymal stromal cells
Source: Cell Tissue Res. 2021 Jul 9;386(2):309–20. doi: 10.1007/s00441-021-03498-5 (PMC8557148; doi:10.1007/s00441-021-03498-5)
Supplement: Supplementary file 1 — Supplementary file1 (DOCX 2286 KB) [file 441_2021_3498_MOESM1_ESM.docx]

**Supplementary information**

**Supplementary Figure S1. Schematic representation of study set up** (generated with Servier Medical ART:SMART).

**
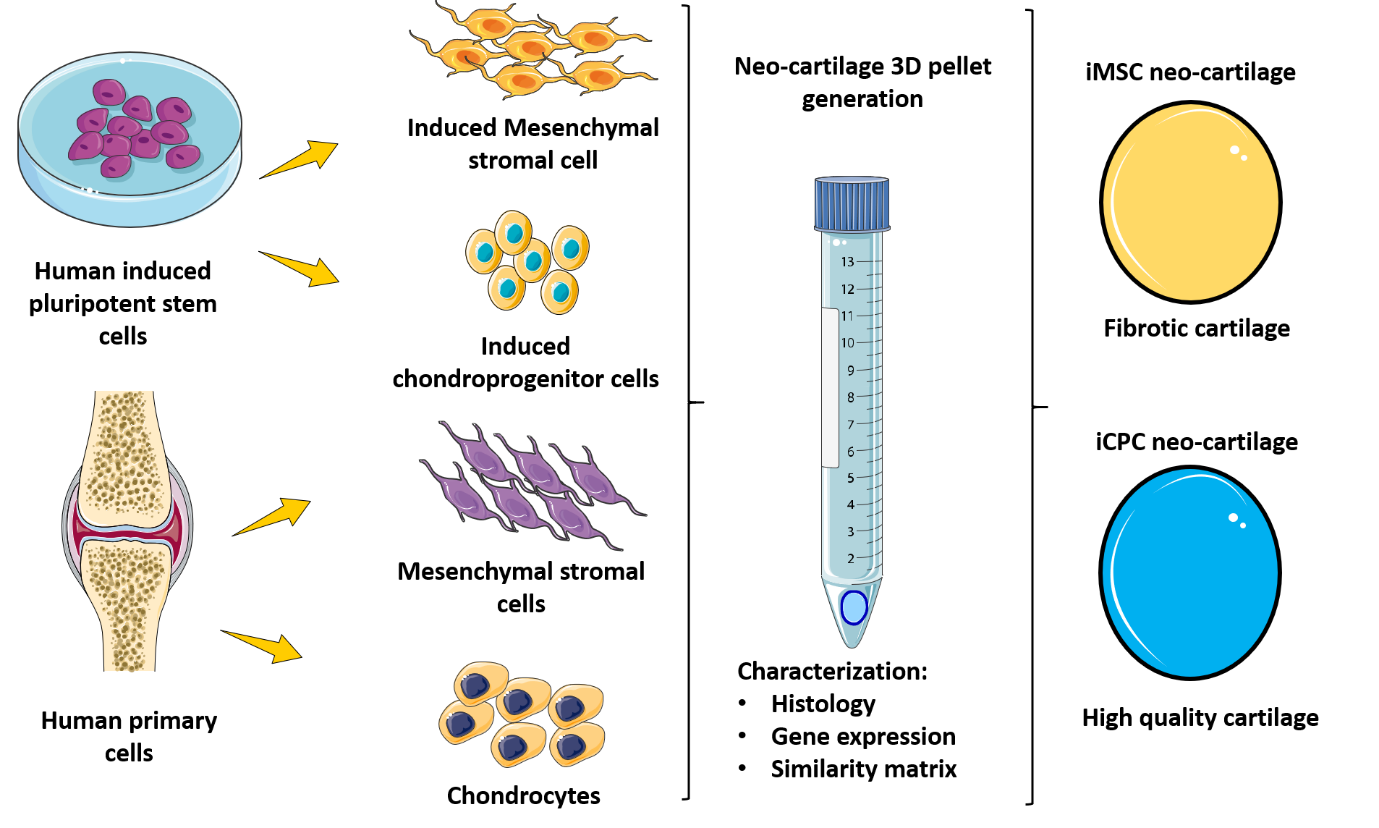
**

**Supplementary Figure S2. Characterization of generated hiPSC control lines.** a) Bright field microscopy image of hiPSC colonies. b) Immunofluorescent staining for NANOG, TRA-1-60, and TRA-1-81 (red), and SSEA-4 and OCT-4 (green) as indicated in the image. Nuclei are stained with Dapi (blue). c) Karyotype demonstrating absence of chromosomal abnormalities. d) Expression of CD31, b3-Tubulin, and AFP, as indicated in the image, upon spontaneous differentiation into the three different lineages (mesoderm, ectoderm, and endoderm, respectively). Control line hiPSC-004: a’, b’’, b’’’, d’’’, d’’’’, d’’’’’; control line hiPSC-030: a, b, b’, d, d’, and d’’.


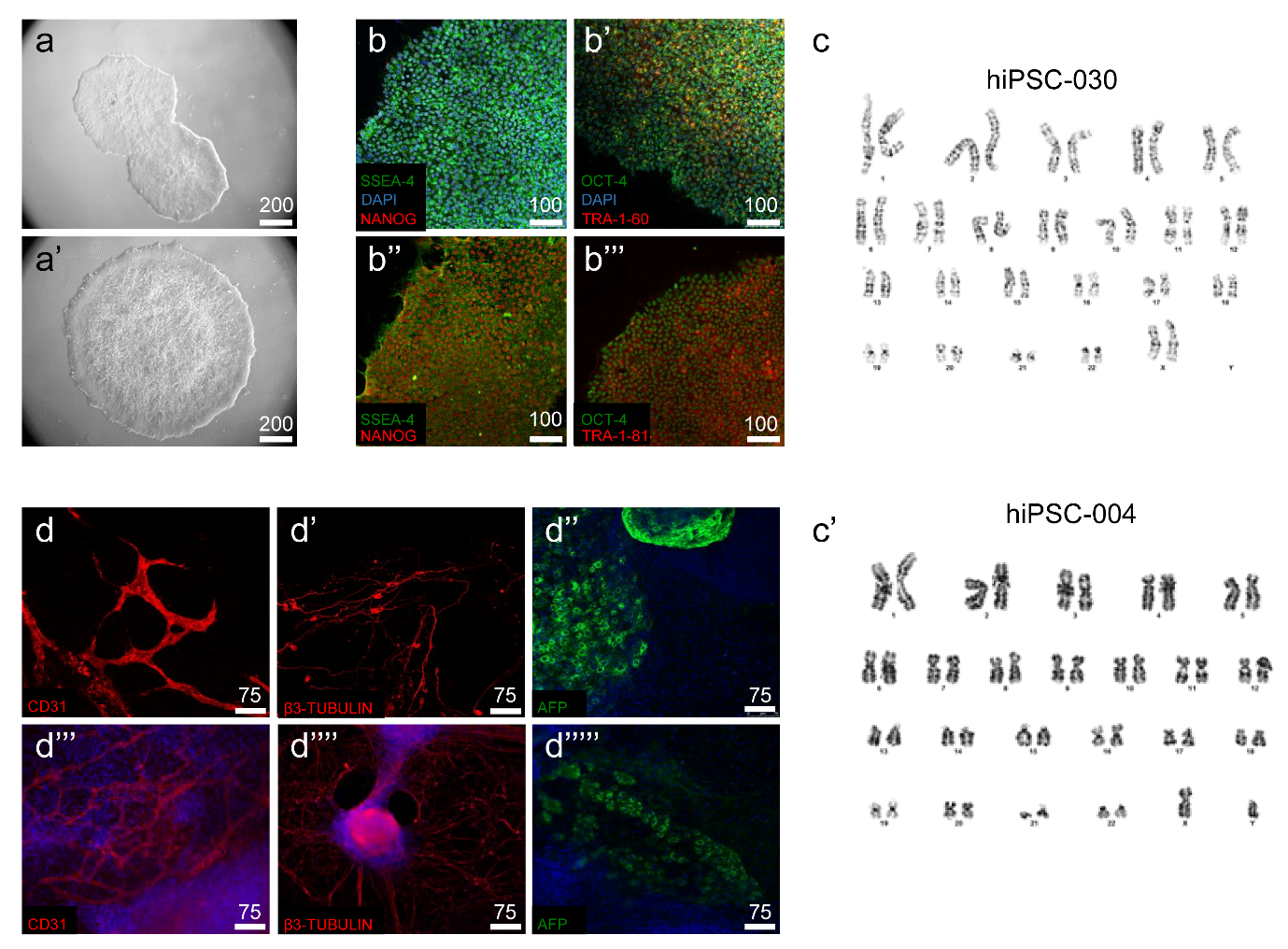


**Supplementary Table S1. Sequences of primers used for RT-qPCR.**

|  | **Primer Sequences** | |
| --- | --- | --- |
| **Matrix Genes** | **Fwd** | **Rvs** |
| *ACAN* | 5'AGAGACTCACACAGTCGAAACAGC-3' | 5'-CTATGTTACAGTGCTCGCCAGTG-3' |
| *COL2A1* | 5'-CTACCCCAATCCAGCAAACGT-3' | 5'-AGGTGATGTTCTGGGAGCCTT-3' |
| *COL1A1* | 5' - GTGCTAAAGGTGCCAATGGT-3' | 5' -ACCAGGTTCACCGCTGTTAC -3' |
| *COL10A1* | 5'-GGCAACAGCATTATGACCCA-3' | 5'-TGAGATCGATGATGGCACTCC-3' |
| **Hypertrophy genes** | **Fwd** | **Rvs** |
| *ADAMTS5* | 5'-CGTGTACTTGGGCGATGACA-3' | 5'-CTGTTGTTGCACACCCCTCT-3' |
| *MMP13* | 5'-TTGAGCTGGACTCATTGTCG-3' | 5'-GGAGCCTCTCAGTCATGGAG-3' |
| *EPAS1* | 5'-ACAGGTGGAGCTAACAGGAC-3' | 5'-CCGTGCACTTCATCCTCATG-3' |
| *WWP2* | 5'-CACATGTGTCTCCTGGTCCC-3' | 5'-GGCAGGGGAAGTGTGCATAT-3' |
| *ALPL* | 5'-CAAAGGCTTCTTCTTGCTGGTG-3' | 5'-CCTGCTTGGCTTTTCCTTCA-3' |
| **Chondrogenesis genes** | **Fwd** | **Rvs** |
| *SOX5* | 5’-CCTCAAAGCCTCTGTCCCAG-3’ | 5’-TGCCTTGGTGACAGCATCAT-3’ |
| *SOX6* | 5’-AACAACGGCAGCAAATGGAC-3’ | 5’-TGGATCTGTTGCTGCAGGAG-3’ |
| *SOX9* | 5'-CCCCAACAGATCGCCTACAG-3' | 5'-CTGGAGTTCTGGTGGTCGGT-3' |
| *FGFR2* | 5'-TCTCTTCAACGGCAGACACC-3' | 5'-AAAGCAACCTTCTCCCAGGG-3' |
| *NOTCH1* | 5'-AGGACTGCAGCGAGAACATT-3' | 5'-GCAGTAGAAGGAGGCCACAC-3' |
| *NOTCH3* | 5'-GTGGATGGCGTCAACACCTA-3' | 5'-CTGCAGCTGACACTCATCCA-3' |
| *SMAD3* | 5'-GCCCCTTTCAGGTAACCGTC-3' | 5'-GAAGCGGCTGATGCTCCTTA-3' |
| *SMAD7* | 5’- CGAATTATCTGGCCCCTGGG-3’ | 5’-TCCCCACTCTCGTCTTCTCC-3’ |
| *GDF5* | 5'-GACATGGTCGTGGAGTCGTG-3' | 5'-CCCCTCTGTGATTCCAGGAGT-3' |
| *PRG4* | 5'-AAAGTCAGCACATCTCCCAAG-3' | 5'-GTGTCTCTTTAGCGGAAGTAGTC-3' |
| *NFAT5* | 5'-AGGCCTGCAGAGTAACTGGA-3' | 5'-CCGCCAGTGTCATGTTGTTG-3' |
| **Housekeeping genes** | **Fwd** | **Rvs** |
| *GAPDH* | 5'-TGCCATGTAGACCCCTTGAAG-3' | 5'-ATGGTACATGACAAGGTGCGG-3' |
| *ARP* | 5'-CACCATTGAAATCCTGAGTGATGT-3' | 5'-TGACCAGCCGAAAGGAGAAG-3' |

**Supplementary Table S2. Similarity tables for the different samples included in the current study.**

**
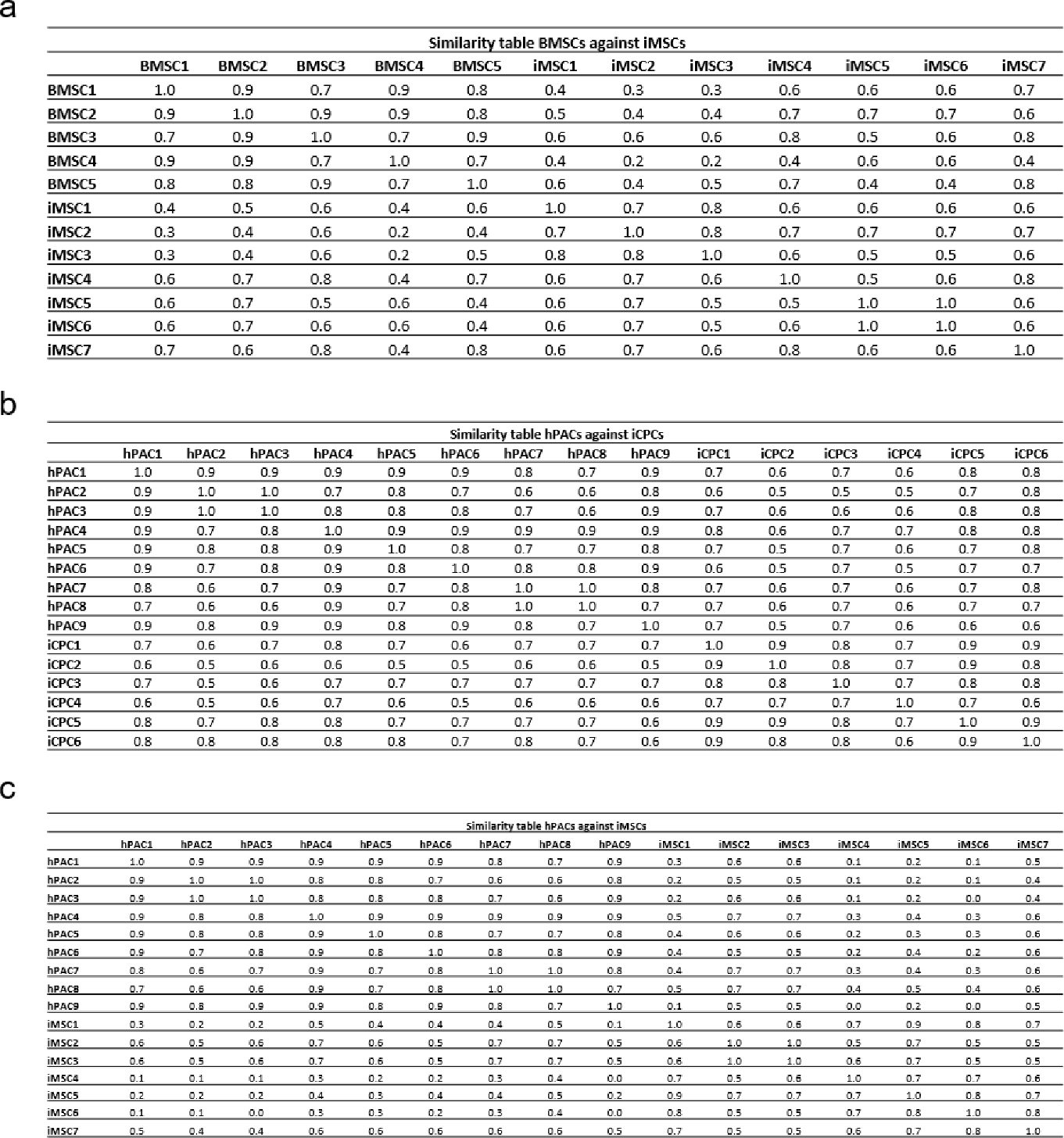
**

**Supplementary Table S3. Differences in gene expression between hiCPC-derived neo-cartilage at week 3 and 5.**

| **hiCPCs week 3 versus week 5** | | | | |
| --- | --- | --- | --- | --- |
| **Matrix genes** | **Fold change** | **Beta** | **SE** | **P value** |
| *ACAN* | -5.1 | -2.8 | 1.7 | 1.0E-01 |
| *COL2A1* | -4.7 | -2.7 | 1.6 | 9.3E-02 |
| *COL1A1* | -1.2 | -0.3 | 0.8 | 7.5E-01 |
| *COL10A1* | -2.4 | -1.6 | 1.1 | 1.5E-01 |
| **Hypertrophy genes** | **Fold change** | **Beta** | **SE** | **P value** |
| *ADAMTS5* | -3.4 | -2.1 | 1.3 | 9.4E-02 |
| *MMP13* | -1.2 | -0.3 | 2.0 | 8.9E-01 |
| *EPAS1* | -5.0 | -2.8 | 1.7 | 1.1E-01 |
| *WWP2* | -1.1 | -0.2 | 0.4 | 5.4E-01 |
| ***ALPL*** | **4.3** | **2.5** | **1.1** | **1.8E-02** |
| **Chondrogenesis genes** | **Fold change** | **Beta** | **SE** | **P value** |
| *SOX5* | -1.5 | -0.7 | 0.5 | 1.7E-01 |
| *SOX6* | 2.0 | 1.2 | 1.4 | 3.7E-01 |
| *SOX9* | -1.1 | -0.2 | 0.8 | 8.0E-01 |
| *FGFR2* | -1.4 | -0.6 | 0.7 | 3.4E-01 |
| *NOTCH1* | 1.0 | 0.0 | 0.6 | 9.6E-01 |
| *NOTCH3* | 1.4 | 0.5 | 0.3 | 6.5E-02 |
| *SMAD3* | 1.5 | 0.7 | 1.0 | 4.4E-01 |
| *SMAD7* | 3.2 | 2.0 | 1.4 | 1.5E-01 |
| *GDF5* | -1.4 | -0.6 | 1.2 | 6.2E-01 |
| *PRG4* | -1.3 | -0.4 | 0.7 | 5.0E-01 |
| *NFAT5* | -1.3 | -0.5 | 0.4 | 2.1E-01 |

(significant differential expression depicted in bold)
